# Supplementary material for: The interactome of intact mitochondria by cross-linking mass spectrometry provides evidence for coexisting respiratory supercomplexes
Source: Mol Cell Proteomics. 2017 Dec 8;17(2):216–32. doi: 10.1074/mcp.RA117.000470 (PMC5795388; doi:10.1074/mcp.RA117.000470)
Supplement: Supplemental Data [file supp_RA117.000470_133922_0_supp_23978_fzffwf.zip › spectra_annotation/mito_spectra_annotation/33-2-1-1-6-1.pdf]

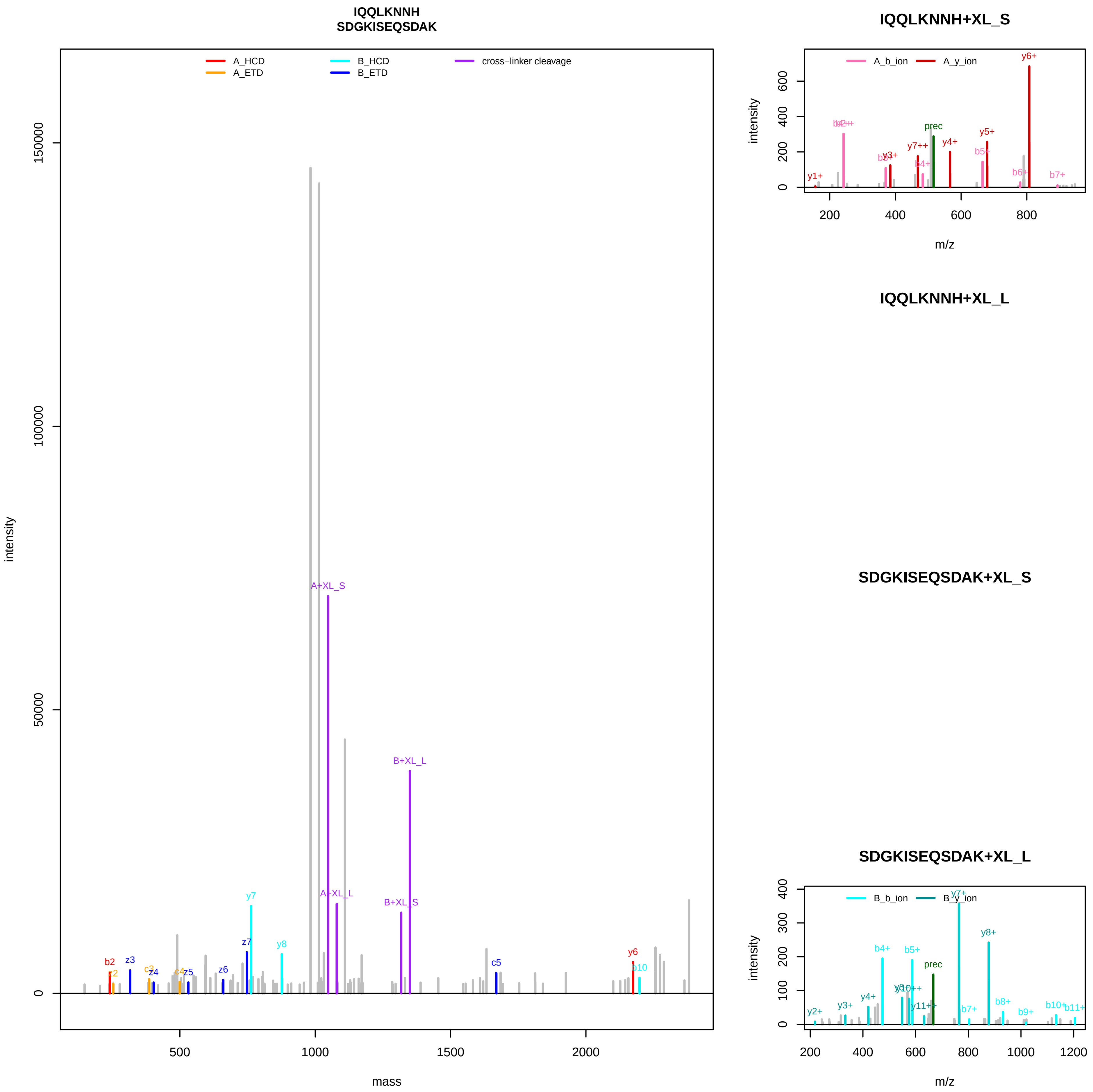
IQQLKNNH
SDGKISEQSDAK
A_HCD
A_ETD
B_HCD
B_ETD
cross−linker cleavage
intensity
mass
150000
100000
50000
0
500
1000
1500
2000
A+XL_S
A+XL_L
B+XL_L
B+XL_S
b2
z3
z4
c3
z5
z6
z7
y7
y8
c5
y6
b10
IQQLKNNH+XL_S
A_b_ion
A_y_ion
y6+
prec
y5+
y4+
y7++
y3+
y1+
b5+
b6+
b7+
b4+
m/z
IQQLKNNH+XL_L
SDGKISEQSDAK+XL_S
SDGKISEQSDAK+XL_L
B_b_ion
B_y_ion
y7+
y8+
b4+
b5+
prec
y10++
y11++
y4+
y3+
y2+
b7+
b8+
b9+
b10+
b11+
